# High-risk behaviors and factors for HIV and sexually transmitted infections among transgender people in Gaborone, Botswana: results from a national survey

**DOI:** 10.11604/pamj.2022.41.128.32430

**Published:** 2022-02-14

**Authors:** Keatlaretse Siamisang, Bornapate Nkomo, Kemmonye Kusi, Dorcus Kanyenvu, Mooketsi Molefi

**Affiliations:** 1 Department of Family Medicine and Public Health, University of Botswana, Gaborone, Botswana,; 2 Department of Health Services Management, Ministry of Health and Wellness, Gaborone, Botswana

**Keywords:** Transgender, key populations, HIV, Botswana, sexually transmitted infections, high-risk behaviors

## Abstract

**Introduction:**

key populations and transgender people in particular are at a high risk of HIV infection. However, very little is known about risk behaviors of transgender people in Botswana. The aim of this study was to determine the prevalence of high-risk behaviors for HIV and sexually transmitted infections (STIs) among transgender people in Botswana.

**Methods:**

data from the Botswana 2017 Biological and Behavioral Surveillance Survey of HIV/STIs among select key populations (BBSS-2) was used. The cross sectional survey documented behavioral risk factors for these infections. This paper only focused on the analysis of the transgender data. Descriptive analysis was done with IBM Statistical Software for the Social Sciences (SPSS) version 24.

**Results:**

there were 56 transgender people identified of which 12 (21.4%) were transgender women. The median age was 24 (interquartile range (IQR) 22-28). Among transgender women, 2 (16.7%) reported concurrent sexual partners and 9 (75%) reported condom use at last intercourse. However, only 7 (58.3%) reported consistent lubricant use. About 45% of the respondents did not know the HIV status of their last male partner. Only one of the transgender women reported intercourse with at least 1 female in the last month. About a third reported that they had STI symptoms in the past year. Alcohol use was reported in 50% of respondents while 83% had disclosed gender identity and had been accepted by their families. However, 25% reported discrimination by a healthcare worker.

**Conclusion:**

the high-risk behaviors were frequent among transgender women. This study underlines the need for sustained efforts to reach this key population.

## Introduction

Transgender refers to individuals who do not conform to the traditional definitions of sex. According to world health organization (WHO), transgender refers to persons “whose gender identity or expression does not conform with the expectations traditionally associated with the sex assigned at birth” [1, 2]. The individuals may or may not undergo genital alteration surgery. Transgender women are people who identify as women or other feminine gender, but who were assigned male sex at birth. On the other hand, transgender men are people who were assigned female sex at birth but who identify as men [3]. Transgender people, especially transgender women (TGW) are at a particularly high risk of HIV infection. Transgender women are therefore considered a key population [2]. Globally, there is a high HIV burden among transgender people. In a recent systematic review, the prevalence of HIV and STIs was high among transgender women and men. The HIV prevalence was 0-49.6% among transgender women and 0-8% among transgender men [1]. In another systematic review and meta-analysis, the overall HIV prevalence was 19.9% among transgender women and 2.5% among transgender men. Significantly, transgender women were 66 times more likely to be HIV positive than other individuals over the age of 16 years. On the other hand, transgender men were about 7 times more likely to be HIV infected than other individuals [4]. In sub-Saharan Africa, the HIV prevalence is high among transgender people. The overall HIV prevalence was 43% in a South-African cohort of men who have sex with men and transgender women. In this study, the annual HIV incidence was 6.2% [5]. In a Kenyan study, the HIV incidence among men who have sex with men and transgender women was 20.6 per 100 person years [6]. In a Ugandan study, the HIV prevalence among transgender men was 4% [7]. These studies demonstrate high HIV prevalence and incidence among transgender people globally and in sub-Saharan Africa.

The high prevalence has been attributed to high-risk behaviors including unprotected anal intercourse, STI coinfection and commercial sex work. In some settings, unsafe injection practices are an additional risk factor for HIV infection in this population [1, 4]. In addition to the individual risk factors, transgender people face significant barriers to HIV services. These include discriminatory policies, healthcare systems sigma and criminalization [8, 9]. Botswana has one of the highest HIV prevalence rates in the world and is second only to Swaziland in this regard [10]. According to UNAIDS 2020 data, there were 380,000 people living with HIV and 9,500 new infections in Botswana in 2019. Furthermore, the HIV prevalence was 20.7% among people aged between 15 and 49 years [11]. The national HIV response has mainly focused on the general population, with limited specific interventions for key populations [12]. It was only in 2012 when key populations were recognized as a target group for focused interventions in the national response [13]. Sexually transmitted infections cause significant morbidity and can strain limited health resources. When left untreated, STIs can cause infertility, acute illness and death [14]. There is increased risk of HIV acquisition and transmission in the context of STI infection [15, 16]. They therefore require a robust public health response [17]. Indeed, STI management is a critical component of successful HIV control programs [15]. World Health Organization (WHO) recognizes that a sustainable HIV response cannot be achieved without addressing the needs of key populations. This cannot be overstated in Botswana where HIV prevalence is high [10, 18].

Across the globe, key populations have borne the brunt of HIV and sexually transmitted infections. Not only are they disproportionately affected by HIV and other sexually transmitted infections, but they also face stigma, discrimination and threat of criminal prosecution [19]. These are significant barriers to high quality care. Key populations are therefore important targets for an effective public health response [17]. There is paucity of evidence on the high-risk behaviors for HIV and sexually transmitted infections (STIs) among key populations in Botswana. This is particularly true for transgender people. Knowledge on the prevalence of high risk behaviors of sexually transmitted infections among key populations in general and transgender people in particular would help guide the ministry of health and wellness´ response. Focusing to prevention and treatment of STIs in transgender people is not just important for their own health; it may also reduce the transmission of STIs and HIV to the general population who inevitably interact with them [20]. Controlling STI transmission among high-risk individuals is a cost-effective way of controlling the HIV epidemic [12]. Indeed, the Global Fund´s strategy for reducing HIV mortality emphasizes investing in areas with high potential impact and strong value for money [21]. Therefore, the aim of this study was to determine the prevalence of high-risk behaviors for HIV and STIs among transgender people in Botswana.

## Methods

**Study design:** data from the Botswana 2017 Biological and Behavioral Surveillance Survey of HIV/STIs among select key populations (BBSS-2) was used. The BBSS-2 was a cross sectional survey of HIV and STIs among selected key populations in Botswana. The survey set out to estimate the prevalence of HIV and other STIs, incidence of HIV as well as the risk profiles of key populations in Botswana [22]. The 2017 BBSS-2 was the second national surveillance of HIV and STIs among key populations in Botswana following up on the 2012 survey. The select key populations targeted were men who have sex with men, female sex workers, transgender people and injection drug users. This paper only focused on the analysis of the prevalence of behavioral risk factors for HIV and STIs among transgender people. The questions on high-risk behaviours in the BBSS-2 were only applicable if participants reported sexual intercourse with a man. Therefore most of the questions were not applicable to most transgender men because they had never engaged in sexual intercourse with a man.

**Study sites:** data for BBSS-2 was collected in 2017 in 5 districts namely greater Gaborone, greater Francistown, Palapye, Maun and Kasane. However, transgender data was only available for Gaborone. Gaborone is the capital and largest city in Botswana and is located in the southeastern part of the country.

**Study population:** this analysis included transgender men and women. In the BBSS-2, a transgender person was defined as anyone 16 years or older whose gender identity was different from the biological sex assigned to them at birth.

**Sample size and selection of subjects:** all the transgender men and women in the BBSS-2 were included in this study. In the BBSS-2, the calculated sample size for transgender population was 320 across 5 districts. The actual sample size was only 56 people, who were all from Gaborone. The small sample size was due to the fact that this is a hard-to-reach population in Botswana. Indeed, this was the first survey on this population in Botswana. Transgender people were not included in the 2012 BBSS-1. Respondent driven sampling (RDS) was used for transgender people in BBSS-2. This was appropriate as this is a hard-to-reach population. The methodology has been used in previous studies [23-25]. RDS is considered to be a quasi-probability sampling method and is clearly superior to non-probability sampling methods such as convenience sampling. It combines a snowballing sampling technique with a mathematical model. It also can reach more hard-to-reach populations who could be otherwise grossly underrepresented in probability sampling methods [25]. Initial seeds were to be identified in each district with the help of local partners. These individuals then went on to identify up to 4 more participants, who in turn identified a maximum of four people. For this paper, all the 56-transgender people in BBSS-2 were included [26].

**Data analysis:** after data cleaning, IBM Statistical Software for the Social Sciences (SPSS) software version 24 was used for data analysis. Due to the small sample size, analysis was limited to simple descriptive analysis. Frequencies and percentages were used to summarize categorical data, while numeric data was summarized with medians and interquartile ranges. Where there was no response (missing data), it was presented as no response.

**Ethical issues and protection of human subject:** ethical review for this analysis was sought and obtained from the University of Botswana Office of Research and Development and from the Ministry of Health and Wellness (permit No HPDME 13/18/1). This study only used data already collected by the Ministry of Health and Wellness and its partners. There were no further attempts to contact any of the participants, and they were not subjected to any additional procedures or activities that may harm or cause them any degree of discomfort or inconvenience.

## Results

There were 56 transgender people identified in the BBSS-2 of which 44 (78.6%) were transgender men and 12 (21.4%) were transgender women (Table 1). The median age was 24 years (IQR 22-28). The highest education attained was tertiary in 44.6% of participants and middle (junior secondary school) in only 17.9% of participants. About 43% of the participants had a monthly income of less than 1000 Botswana Pula (BWP) or 91 United States dollars (USD) while 3.6% had a monthly income of over 10,000 BWP (910 USD). Baseline characteristics are stratified according to whether participants were transgender men or women in Table 2. The age ranges were 22-43 and 18-34 for transgender women and transgender men, respectively. About 82% of transgender men self-identified as homosexual, compared with 25% of transgender women. About two-thirds of transgender women had attained higher education, compared to 38.6% of transgender men. Almost 30% of transgender men reported living with a regular partner or spouse, compared to only 16.7% of transgender women. The sexual history and high-risk behaviors of transgender women are presented in Table 3. Among transgender women, 16.7% reported concurrent sexual partners at the time of the interviews. The number of male partners, the participants had intercourse within the preceding six months ranged from 1 to 11 with a median of 2. Three-quarters of the participants reported they had used a condom the last time they had intercourse with a male partner.

**Table 1:** baseline characteristics for transgender persons in Gaborone

Variable	Number (%)
**Age, median (inter-quartile range)**	24 (22-28)
Transgender men	44 (78.6%)
Transgender women	12 (21.4%)
**Self identified sexual orientation**	
Bi-sexual	3 (5.4)
Homosexual	39 (69.9)
Heterosexual	12 (21.4)
Do not know	2 (3.6)
**Highest education level attained**	
Higher	25 (44.6)
Middle (junior secondary school)	10 (17.9)
Secondary (senior secondary school)	21 (37.5)
**Employment status**	
Unemployed	23 (41.1)
Employed	33 (58.9)
**Monthly income**	
None	12 (21.4)
Less than BWP1000	12 (21.4)
Between BWP1000 and BWP2000	19 (33.9)
Between BWP 2000 and BWP 5000	7 (12.5)
Between BWP 5000 and BWP 10000	4 (7.1)
Over BWP 10000	2 (3.6)

**Table 2:** baseline characteristics for transgender men and women in Gaborone

Variable, no (%)	Transgender men (n=44)	Transgender women (n=12)
**Age, mean (standard deviation)**	24.4 (4.1)	26.3 (5.8)
**Age, range**	18-34	22-43
**self identified sexual orientation**		
Bi-sexual	21 (4.5)	1 (8.3)
Homosexual	36 (81.8)	3 (25.0)
Heterosexual	5 (11.4)	7 (58.3)
Do not know	1 (2.3)	1 (8.3)
**Highest education level attained**		
Higher (tertiary)	17 (38.6)	8(66.7)
Middle (junior secondary school)	9 (20.5)	1(8.3)
Secondary (senior secondary school)	18 (40.9)	3 (25.0)
**Employment status**		
Unemployed	18 (40.9)	5 (41.7)
Employed	26 (59.1)	7 (58.3)
**Monthly income**		
None	12 (27.3)	0 (0)
Less than BWP1000	8 (18.2)	4 (33.3)
Between BWP1000 and BWP2000	14 (31.8)	5 (41.7)
Between BWP 2000 and BWP 5000	6 (13.6)	1 (8.3)
Between BWP 5000 and BWP 10000	4 (9.1)	0 (0)
Over BWP 10000	0 (0)	2 (16.7)
**Who participants live with at home**		
Lives alone	7 (15.9)	4 (33.3)
Family	20 (45.5)	6 (50.0)
Spouse/regular partner	13 (29.5)	2 (16.7)
Other	4 (9.1)	0 (0)

**Table 3:** general sexual history of transgender women in Gaborone

Variable	Number (%)
Forced first sexual intercourse with a man	1 (8.3)
History of forced sex with a male partner in the last 6 months	1(8.3)
No of male sexual partners with whom the participants had receptive anal sex in the last 6 months, median (IQR)	2 (1-4)
No of male sexual partners with whom the participants had receptive anal sex in the last 6 months, range	1-11
Always used a condom with a male partner in the last 6 months	6 (55%)
History of being forced to not use condoms	2 (16.7)
Current concurrent sexual partners	2 (16.7)
Use of a condom at last anal intercourse with a man	9 (75.0)
Did not know HIV status of last male partner	5 (45%)
Consistent lubricant use during sex in the last 6 months	7 (58.3)
History of intercourse with at least 1 female in the last 6 months	1 (8.3)
History of STI symptoms in the past year	4 (33.3)

However, only 55% reported using a condom at every sexual encounter in the preceding 6 months. In addition, 58.3% of transgender females reported consistent lubricant use during sexual intercourse in the last 6 months. About 45% of the respondents did not know the HIV status of their last male partner. One of the transgender women reported a history of forced sexual intercourse with a man that had happened within 6 months of the interviews. Only one of the transgender females reported intercourse with at least 1 female in the last month. About a third of the transgender women reported that they had STI symptoms in the past year. In terms of alcohol use, 50% of transgender women reported drinking alcohol at least once a week. Only 16.7% reported not taking alcohol at all, and one participant reported taking alcohol every day. About 41.5% of the participants reported that they had never had sexual intercourse while intoxicated, while one person reported she had intercourse while intoxicated 2 or 3 times a week. About 58% of transgender thought that anal sex is more risky than vaginal sex for acquiring HIV, while 33.3% thought the 2 are equally risky. About two-thirds of transgender women reported thought that it was very easy to obtain condoms or lubricants (Table 4). Acceptance, stigma and discrimination based on gender identity or sexual orientation are presented in Table 5. About 83% of the transgender women had disclosed gender identity and sexual orientation and had been accepted by their families. A quarter of the transgender women reported discrimination in a healthcare facility because of their gender identity or sexual orientation. The same proportion reported they had been refused housing. One participant reported being discriminated against in a workplace or being fired because of their sexual orientation. About a third of the respondents had been discriminated in school or university, while 16.7% reported a history of gender based violence. However, none of the respondents reported harassment by law enforcement agents based on their gender identity or sexual orientation.

**Table 4:** transgender women knowledge and history of STIs

Variable	Number (%)
**Frequency of use of alcoholic drinks**	
Never	2 (16.7)
Less than once a week	2 (16.7)
At least once a week	6(50)
Every day	1(8.3)
No response	1(8.3)
**Frequency of sexual intercourse while intoxicated**	
Never	5 (41.5)
Less than monthly	1 (8.3)
Two or three times a week	1 (8.3)
Weekly	2 (16.7)
No response	3 (25.0)
**Response to whether anal sex is more or less risky than vaginal sex for acquiring HIV/Aids**	
Anal sex is more risky	7 (58.3)
Anal sex is less risky	0 (0)
The two are equally risky	4 (33.3)
No response	1 (8.3)
**Ease of obtaining condoms**	
Very easy	8 (66.7)
Somewhat easy	3 (25.0)
Not easy	0(0)
No response	1 (8.3)
**Ease of obtaining water based lubricant**	
Very easy	8 (66.7)
Somewhat easy	1 (8.6)
Not easy	2 (16.7)
No response	1 (8.3)

**Table 5:** stigma and discrimination among female transgender people

Variable	Number (%)
Discrimination in healthcare facility based on gender identity and/or sexual orientation	3 (25.0)
Refusal of housing based on gender identity and/or sexual orientation	3 (25.0)
Discriminated at a job or being fired based on gender identity and/or sexual orientation	1 (8.3)
Discrimination in a school or university based on gender identity and/or sexual orientation	4 (33.3)
History of suffering gender based violence based on gender identity and/or sexual orientation	2 (16.7)
Harassment by law enforcement agents based on gender identity and/or sexual orientation	0 (0)
Disclosure of gender identity and/or sexual orientation to family	10 (83.3)
Acceptance by family	10 (83.3)

## Discussion

This is a report on the first survey of the behavioral risk factors for HIV and STIs among transgender people in Botswana. The fact that only 56 transgender people and 12 transgender women were identified despite efforts in a national survey demonstrates the difficulty of reaching this population. Analysis of high-risk behaviors and other factors was limited to transgender females, since most transgender men reported no history of sexual intercourse with a man. The BSSS-2 questionnaire was designed such that if a participant had no history of sexual intercourse with a man, no further questions were asked and the interview was stopped. The findings suggest that transgender women in Botswana are at a high risk of HIV infection. Multiple high-risk behaviors were identified. This includes confirmation that members of key populations do have sexual relationships with the general population. Additionally, use of alcoholic drinks was common among the transgender women. Furthermore, a significant proportion of transgender women reported some form of discrimination or stigma related to their sexuality. STIs were also common, with a third of the transgender women reporting a history of STI symptoms in the past year. Both transgender men and women interviewed were relatively young. This may be because younger people are more likely to openly discuss their sexuality. Previous non-probability methods in this setting have tended to have young participants. In a study of HIV prevalence and risk factors among MSM in Malawi, Namibia and Botswana, the median ages of the participants in each of the three countries was 24-26 [27]. Social and legal barriers likely played a role in preventing presentation and interaction of transgender people with the survey personnel. Fear of legal and social repercussions have been cited as a barrier to participation in research by transgender people in Africa [28]. Previous work on key populations in Botswana has also struggled to reach transgender women. In the study of HIV risk in Malawi, Namibia and Botswana only 1/117 (0.9%) MSM in Botswana identified as transgender. Transgender women accounted for 1.3% of the participants in the pooled data [27]. Interventions to reach out to this key population are mandatory if an effective and sustainable HIV response is to be achieved [29].

The frequency of most of the high risk behaviors were generally higher than what has been previously reported for the general population in Botswana. In the 2013 fourth AIDS Impact Survey (BIAS IV), 15.8 % of the participants reported multiple sexual partners, while 81.9% had used a condom at the last sexual encounter [10]. However, the high-risk behaviors were comparable with those of other key populations. Baral *et al*. reported a similar number of male sexual partners in the past 6 months for MSM in Botswana. The median number was 2 with a range of 0 to 24 [27]. However, use of a condom at last anal intercourse in the present study was less frequent than what was reported for MSM in the first (2012) BBSS [13, 30]. The proportion of transgender women who reported sex with a female in the last 6 months was lower than what has been reported in other studies. In a study of HIV incidence among men who have sex with men (MSM) and transgender women (TGW) in sub-Saharan Africa, 61 % of the participants reported having had intercourse with a woman [31]. This much higher proportion is likely related to inclusion of MSM in the analysis. MSM tends to report more sexual relationships with women than transgender women. More than one third of MSM in the first Botswana BBSS reported having sex with a female partner in the last 6 months [13]. A high proportion of the participants in the current study reported that they had disclosed their gender identity and sexual orientation to their families, and their families had accepted all of them. This is an encouraging finding. Rejection by families is a significant barrier to disclosure of sexual identity and orientation in many settings [2]. Only about 17% of the transgender women reported never taking alcohol, compared to 53.2% of the general population in the fourth Botswana AIDS impact survey [10].

Use and abuse of alcohol was common among other key populations in Botswana´s first BBSS (2012). Among the female sex workers, 17.2% reported daily alcohol use while 55.4% taking at least 6 alcoholic drinks before intercourse [12, 13]. Sandfort *et al*. reported similar proportions of intercourse while intoxicated. Half of participants in this study reported sexual intercourse under the influence of alcohol [31]. The use of alcohol has been shown to be significantly associated with unprotected anal intercourse and inversely associated with the use of water-based lubricants [32]. Both of these are established drivers of HIV transmission among MSM and TGW [30]. Our findings underline the need to include alcohol abuse prevention and management programs in the HIV prevention efforts for key populations in general and transgender women in particular. Similar to the current study, stigma and discrimination of key populations has been reported in the literature. Baral *et al*. reported similarly high rates of human right abuses affecting MSM in Botswana, with almost 3 in 5 reporting some form of abuse. However, rates of denial of healthcare and housing were lower at 5.2% and 0.85% respectively [27]. Discrimination against men who have sex with men and transgender women is common in sub-Saharan Africa. In the study by Sandfort *et al*. 44.4% of MSM and TGW had experienced homophobia of some kind. A similar proportion had tried to hide their sexuality [31].

Violence against transgender people is also common. In a study of sexual violence in 9 African countries, including Botswana, a significant proportion of transgender women experienced physical and sexual violence. About 73% reported ever experiencing violence, while 44.6% had experienced violence in the past year. This population was generally more affected by violence than transgender men and gender non-conforming individuals [33]. In a South African qualitative cohort, transgender women experienced significant stigma, which had a negative impact on their HIV services utilization [34]. Our study highlights the need for specific interventions for key populations in Botswana. HIV control in key populations is essential for successful HIV programming. Receptive anal intercourse is a well-known risk factor for HIV infection, as is unprotected sexual intercourse without use of water-based lubricants [1, 26]. Interventions need to address these risk factors, including making condoms and water-based lubricants accessible for transgender women. Public health interventions should also be targeted at identifying and treating STIs. Untreated STIs have been associated with increased risk of HIV infection [28]. Our findings also suggest that transgender women may be more likely to either present late or not seek medical treatment for STIs because of the discrimination and stigma associated with their gender identity. This underlines the need to strengthen efforts to reach this population in Botswana.

**Strengths:** despite the small sample size, this study provides very useful information on the transgender population in Botswana. This is the first descriptive study of the high-risk behaviors among transgender people in Botswana and is an important addition to the literature. The paper is based on a national survey with appropriate methodology for this hard to reach population. The study includes both transgender men and women even though most of the analysis focuses on transgender women. The study addresses several key questions in the assessment of risk factors for HIV including sexual history, HIV and STI knowledge and experience of stigma and discrimination. This provides a broad understanding of the HIV and STI risk in this population in Gaborone, Botswana. Future, larger studies can build on these findings and explore these factors further.

**Limitations:** our study has several limitations. Firstly, the sample size was very small especially for transgender women. There were only 12 transgender women in this study. This means that the results have to be interpreted with caution. The study is particularly susceptible to random error. As the risk behaviors were self-reported, misclassification bias through social desirability bias could not be avoided. The small sample size also meant the study was limited to descriptive analysis. This paper only focuses on the high-risk behaviors and does not report the HIV prevalence in this population. The other limitations are related to the cross-sectional nature of the design including recall bias and non-response. Furthermore, there is limited generalizability of the study findings beyond Gaborone, Botswana.

## Conclusion

This study provides an important description of the high-risk behaviours and factors that are associated with HIV infection among transgender people in Botswana. The high-risk behaviors including multiple sexual partners, use of alcohol and unprotected sex were frequent among transgender females. A significant proportion of this key population had also experienced stigma and discrimination. This study underlines the need for sustained efforts to reach out to this key population. Furthermore, public health interventions including promotive and preventive HIV services must be tailored to the needs of transgender people. Interventions must also facilitate an enabling environment for transgender people to access services.

### What is known about this topic

- *Key populations including transgender people are at high risk of HIV infection;*
- *Social and legal barriers limit transgender people’s access to HIV services including promotive, preventive and curative services*.

### What this study adds

- *In this high HIV prevalence country, transgender populations are incredibly hard to reach;*
- *High risk behaviors for HIV and sexually transmitted infections are common among transgender people*.
